# Impact of continuing medical education for primary healthcare providers in Malaysia on diabetes knowledge, attitudes, skills and clinical practices

**DOI:** 10.1080/10872981.2019.1710330

**Published:** 2019-12-31

**Authors:** Shiang Cheng Lim, Feisul Idzwan Mustapha, Jens Aagaard-Hansen, Michael Calopietro, Tahir Aris, Ulla Bjerre-Christensen

**Affiliations:** a Steno Diabetes Center Copenhagen, Gentofte, Denmark; b United Nations University – International Institute for Global Health, Kuala Lumpur, Malaysia; c Department of Public Health, Ministry of Health Malaysia, Putrajaya, Malaysia; d MRC Developmental Pathways for Health Research Unit, Faculty of Health Sciences, University of the Witwatersrand, Johannesburg, South Africa; e Institute for Public Health, Ministry of Health Malaysia, Putrajaya, Malaysia

**Keywords:** Clinical practice, continuing medical education, diabetes, healthcare provider, Malaysia, mixed methods, primary care, Solomon’s four-group design

## Abstract

**Background**: Continuing Medical Education (CME) is a cornerstone of improving competencies and ensuring high-quality patient care by nurses and physicians. The Ministry of Health (MOH) Malaysia collaborated with Steno Diabetes Centre to improve diabetes-related competencies of general physicians and nurses working in primary care through a six-month training programme called the Steno REACH Certificate Course in Clinical Diabetes Care (SRCC).

**Objective**: This impact evaluation aimed to assess the effect of participation of general physicians and nurses in the SRCC in selected public primary healthcare clinics in Kuala Lumpur and Selangor, Malaysia.

**Design**: The quasi-experimental, embedded, mixed-methods study used concurrent data collection and the Solomon four-group design. Participants in an intervention group (Arm 1) and control group (Arm 3) were assessed by pre-and post-test, and participants in separate intervention (Arm 2) and control (Arm 4) groups were assessed by post-test only. Quantitative and qualitative methods were used to assess the effect of the programme.

**Results**: Thirty-four of the 39 participants in the intervention groups (Arms 1 and 2) completed the SRCC and were included in the analysis. All 35 participants in the control groups (Arms 3 and 4) remained at the end of the study period. Significant improvements in diabetes-related knowledge, skills and clinical practise were found among general physicians and nurses in the intervention group after the six-month SRCC, after controlling the pretest effects. No clear changes could be traced regarding attitudes.

**Conclusion**: SRCC participants had significant improvements in knowledge, skills and clinical practice that meet the current needs of general physicians and nurses working in primary care in Malaysia. Thus, SRCC is an effective CME approach to improving clinical diabetes care that can be scaled up to the rest of the country and, with some modification, beyond Malaysia.

## Introduction

The global burden of noncommunicable diseases (NCDs) constitutes a major public health challenge. Diabetes is one of the four major NCDs. The burden of diabetes is highest in low- and middle-income countries, which contain most of the world’s population. These countries have witnessed dramatic increases in the prevalence of diabetes in recent decades [1].

The burden of diabetes continues to increase in Malaysia. Data from the 2015 population-based National Health and Morbidity Survey showed a prevalence of 17.5% among adults aged 18 years and above, corresponding to approximately 3.5 million adults [2]. Consequently, the Malaysian healthcare delivery system is facing increasing pressure to provide quality care to patients with diabetes. Approximately 80% of patients diagnosed with diabetes seek treatment at Ministry of Health (MOH) healthcare facilities, a proportion that is expected to continue to increase [2]. However, the public health sector includes an insufficient number of medical practitioners who are well-trained in evidence-based diabetes care to handle the growing diabetes population [3].

The importance of good diabetes care is well established, and much research has been done on how to improve the quality of diabetes care [4–7]. In addition to early detection and diagnosis, appropriate clinical management and patient engagement, education and empowerment reduce the risk of diabetes-related complications and enable patients with diabetes to maintain a good quality of life. Many studies have examined the effectiveness of quality improvement strategies in diabetes care [8–11]. Interventions such as delegating tasks to nurse practitioners, point-of-care reminders and continuing education have a positive effect on the quality of diabetes care. Studies have also shown the importance of specialised diabetes teams, clinics or services to increasing the quality of care and reducing the risks of diabetes-related complications [10,12,13].

A large focus of addressing gaps in the management of NCDs is building human resource capacity [14,15]. Continuing Medical Education (CME) is a cornerstone of developing competencies and ensuring high-quality patient care. In a synthesis of systematic reviews, Cervero and Gaines (2015) concluded that CME improves physician performance and patient outcomes; however, further studies were needed to examine the implementation of knowledge, skills and attitudes and the impact of contextual and implementation factors on CME [16]. Despite the importance of CME, only a few studies have measured its impact on clinical diabetes practice and patient outcomes in a real-world setting [17–19]. These studies showed varying results [17,19–22]. Studies have also shown that CME is associated with increased satisfaction and better psychosocial wellbeing of diabetes patients [20] and is very well received among participating health care providers (HCPs) [23].

As part of its effort to build diabetes management capacity, MOH Malaysia is collaborating with Steno Diabetes Center Copenhagen educate general physicians and nurses at the primary care clinics about the fundamentals of diabetes care through the Steno REACH Certificate Course (SRCC). SRCC is a competency-based approach that combines e-learning, classroom-based learning and clinic-based group work. The goal is to improve the knowledge, skills and attitudes of participating HCPs in clinical diabetes management, enabling them to provide high-quality diabetes care. The intervention in this study was designed for primary care physicians and nurses with little training in diabetes care and was facilitated by a team of Malaysian HCPs, who were trained to deliver the programme by experts from Steno Diabetes Center Copenhagen.

The goal of this study was to evaluate the impact of the SRCC course on diabetes-related knowledge, skills, attitudes and clinical practice among Malaysian primary care physicians and nurses.

## Materials and methods

### Description of SRCC

**Figure 1.**
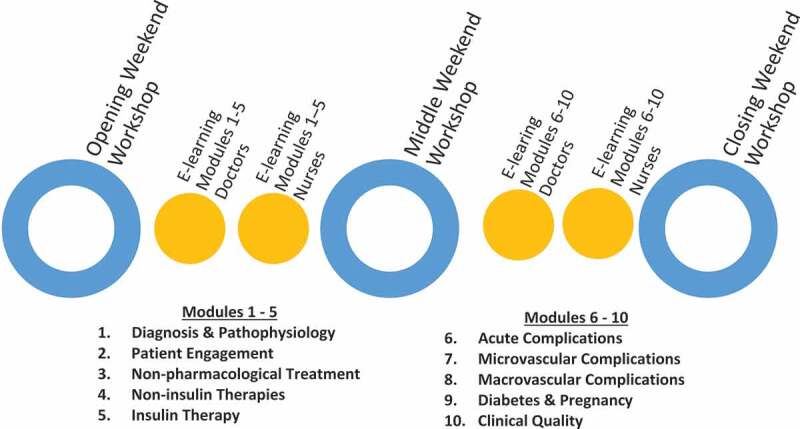
The design of the Steno REACH Certificate Course (SRCC) in clinical diabetes care

The SRCC curriculum had ten modules and was designed to include approximately 50 hours of independent online study and 48 hours of face-to-face classroom time (Figure 1). Separate versions of each module were tailored to the professional roles of general physicians and nurses. E-learning content included all foundational materials delivered in an interactive learning environment. Participants independently completed modules 1 through 5 before reinforcing key learning outcomes through interactive classroom learning activities, repeating the same process for modules 6 through 10.

Figure 1 describes the design of the six months Steno Reach Certificate Course (SRCC), a course with a combination of e-learning and classroom-based group work in three weekend workshops for general physicians and nurses in Malaysia. Ten online modules covered various aspects in diabetes care were designed with two different versions for general physicians and nurses according to their job roles. The SRCC started with an Opening Weekend Workshop and participants were expected to complete the first five modules through self-directed learning before coming to the Middle Weekend Workshop. This experience repeated itself for modules 6 to 10 prior to the Closing Weekend Workshop.n: Number of participants; P: Physicians; N: Nurses

In addition, participants were assigned work-based learning activities to complete between classroom sessions. Work-based learning activities included defining and reflecting on personal learning goals, patient care journaling, discussing articles and cases with clinic peers and reviewing medical records with clinic leaders. Collectively, work-based learning activities required 25 to 30 hours in addition to independent study and classroom time.

### Study design

This study used a quasi-experimental, multi-phase mixed methods approach based on the Solomon four-group design (Table 1). The intervention group in Arm 1 received both a pre-test and a post-test, and three additional groups were included. An intervention group received training and only a post-test (Arm 2), a control group without training received both a pre-test and a post-test (Arm 3), and a control group received a post-test but no training (Arm 4). This design reduces the influence of confounding variables and allows the researcher to assess the effect of pre-testing on measured outcomes.

**Table 1.** Solomon four-group design

Group	Arm	Pre-test	Training	Post-test
Intervention	1	T	X	T
Intervention	2		X	T
Control	3	T		T
Control	4			T

T = Testing condition (Consists of MCQ, OSCE and DAS). In addition, observation and interviews were conducted pre- and post-intervention for Arm 1.

X = SRCC.

### Selection of clinics and participants

The study was conducted at MOH primary health clinics in the states of Kuala Lumpur and Selangor, Malaysia. All MOH health clinics in Selangor and Kuala Lumpur with > 1,000 active diabetes patients (as defined by and registered in the National Diabetes Registry) were included in two-stage stratified random sampling (based on the number of registered active diabetes patients as well as variety of medical services provided by the clinics) to select ten intervention clinics. Selected clinics were randomly assigned to Arm 1 or Arm 2. Control groups were then purposively selected from the remaining primary health care clinics and allocated to Arms 3 and 4 to match their counterpart intervention groups (Arms 1 and 2, respectively) on the basis of mean HbA_1C_ for 2016, which was obtained from clinical audit data and used as a proxy indicator of diabetes care quality.

The Family Medicine Specialist (FMS) at each clinic, who was also the administrative and clinical head, selected participants in the two intervention groups. Control group participants were then purposively selected on the basis of their clinical experience with diabetes patients, with the aim of matching as closely as possible the clinical experience of intervention group participants. Control group participants were offered enrolment in an SRCC class after the study completion.

### Data collection

Both quantitative and qualitative methods were applied to achieve the study aims. Changes in diabetes-related knowledge, skills and attitudes among participants were respectively measured by multiple-choice questionnaire (MCQ), objective structured clinical examination (OSCE) and the Diabetes Attitude Scale, third version (DAS-3) questionnaire. Quantitative pre-intervention assessments were conducted for the pre-test groups (Arms 1 and 3) from June to August 2016, before the start of the SRCC training programme. All participants from all arms were required to participate in the quantitative post-intervention assessments in March 2017.

Application of skills and changes in clinical practice were assessed by triangulating qualitative methods that included observations and interviews with participants from the Arm 1 clinics before and after the intervention.

#### MCQ

The multiple choice assessment of clinical diabetes-related knowledge was developed by a team comprising endocrinologists and diabetes nurses, based on a review of the programme learning objectives and curriculum for physicians and nurses. The physicians’ version included 79 questions in English, and the nurses’ version included 67 questions and was available in both English and Malay language.

#### OSCE

The OSCE was developed by the same team of endocrinologists and diabetes nurses to assess participants’ technical clinical skills and soft skills (patient engagement and empowerment and communication) in managing patients with diabetes, based on SRCC learning objectives and curriculum. The OSCE relied on well-trained simulator patients located at nine stations for physicians and eight stations for nurses, each of which measured skills pertaining to a different domain of diabetes care. At each station, participants were asked to perform a limited number of tasks related to diabetes care management within 15 minutes. Two trained examiners, who were FMS for physicians and senior diabetes educators for nurses, at each station assessed participants’ performance on a scale (‘fail’, ‘borderline’, ‘pass’, ‘good’, ‘very good’). The OSCE was conducted in the Malay language.

#### DAS-3

The DAS-3 is a previously validated general measure of diabetes-related attitudes developed by the University of Michigan Diabetes Research and Training Center [24]. The DAS is designed to evaluate changes in attitudes related to professional education programmes that include the domains measured by the five DAS-3 subscales [24]: the need for special training in education, seriousness of type 2 diabetes, the overall value of tight glucose control in diabetes care, psychosocial impact of diabetes on patients, and patient autonomy. Participants completed the English version of the DAS-3.

#### Direct clinical observation of participants and clinics in Arm 1

Finally, to assess the translation of new knowledge into clinical practice, an experienced clinician on the research team conducted direct observations of the clinical practice of the 23 HCPs from the six participating clinics in Arm 1. The researcher spent an average of two working days using an observation guide to assess each HCP for the level of diabetes-related clinical proficiency demonstrated in each clinical consultation. Data from each observation session consisted of a qualitative assessment of the clinical skill and assignment of one of five proficiency levels (ranging from fundamental awareness to novice, intermediate, advanced and expert) based on criteria established by the National Institutes Health Proficiency Scale [25]. In addition, the soft skills were assessed based on a four-step rating (ranging from poor to moderate, good and excellent).

#### Semi-structured interviews with SRCC participants

A total of 46 pre- and post-intervention in-depth interviews were conducted with all participants in Arm 1 clinics to better understand their history with diabetes-related training, typical clinical encounters with people with diabetes and daily clinic life, as well as the extent to which they applied new knowledge, skills and attitude in clinical practice. The interviews followed a semi-structured interview guide and were audio-recorded.

### Data analysis

#### Analysis of MCQ, OSCE and DAS

Quantitative data analysis was performed using the IBM SPSS version 21.0. Data were cleaned, and all variables were tested for normality with measures of skewness and kurtosis. Descriptive data analysis included mean, percentage and standard deviation for each variable. The association between categorical variables such as ethnicity and sex among the four arms was examined with chi-square tests. Mean differences in continuous variables were determined by one-way analysis of variance (ANOVA) when data were normally distributed and by the Kruskal-Wallis test when data were not normally distributed.

As suggested by Braver and Braver [26], a 2 × 2 factorial ANOVA was conducted on the post-test scores of knowledge, attitude and skills to assess the two main effects, i.e, pretest versus no pretest and intervention versus control. When an interaction effect with pretesting is observed in the 2 × 2 factorial ANOVA, further analysis is conducted among the pretested groups and un-pretested groups by using the main effect test. Significant findings for both pretested and un-pretested groups indicates an intervention effect is observed. Conversely, significant finding in only the pretested group indicate that the effect of the intervention is moderated by pretest exposure.

If no significant interaction effect of pretesting is detected, analysis then proceeds to main effect testing, e.g., intervention vs control. A significant result indicates an intervention effect and no further analysis is required. In the event of nonsignificant findings for the main effect test, repeated measures ANOVA is applied to compare pre-and posttest scores in the two pretested groups. A significant result indicates an intervention effect, and a nonsignificant result indicates the need to test the intervention effect in the non-pretested groups, using t-test. A statistically significant result indicates an intervention effect. A result that is not statistically significant indicates the need to combine the results of the repeated measures ANOVA in the pretested groups and t-test in the un-pretested groups in a meta-analysis, using the following formula to conclude the intervention effect:

$$Z_{meta}=\left(Z_{p1}+Z_{p2}\right)/\sqrt{2}$$

where Z_p1_ is the z value corresponding to the *p*-value of repeated measures ANOVA for the pre-tested groups and Z_p2_ corresponds to the *p*-value of the two-tailed t-test for the un-pretested groups.

#### Analysis of observations and interviews

Field notes were prepared during the observations using predetermined scales for assessment of clinical and soft skills. Changes in the two skill domains were analysed by repeated measures ANOVA.

Audio recordings of interviews with participants from Arm 1 were transcribed. After familiarising themselves with the interview transcripts, the researchers conducted the coding process through Atlas.ti 7 software (ATLAS.ti Scientific Software Development GmbH, Berlin), using predefined codes that were consistent with the objectives of the SRCC and the semi-structured interview guide. In addition, the number of participants who reported applying new knowledge and skills in diabetes care and other clinical areas was tabulated. Relevant quotations from interviews were extracted to support the findings.

## Results

### Participant characteristics

**Figure 2.**
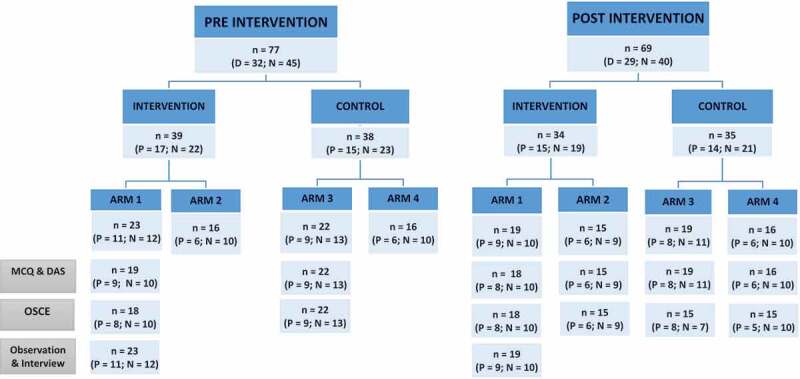
Distribution of participants in pre- and post-intervention by Arm and type of assessment

Thirty-four of 39 participants in the intervention group and 35 of 38 participants in the control group remained at the end of the study period (Figure 2). Eight participants dropped out of the study due to changes in job placement, being transferred to another clinical setting and personal commitments such as childbirth or enrolment in other training courses.

Figure 2 describes the total number of participants, both physicians and nurses in the intervention (Arm 1 and 2) and comparison (Arm 3 and 4) groups and their participation in the pre and post intervention assessments, either quantitative assessments (Multiple Choice Questionnaire (MCQ), Objective Structured Clinical Examination (OSCE) and Diabetes Attitude Scale (DAS) questionnaire) or qualitative assessments (observations and interviews).

Only 33 participants in the intervention group completed the post-intervention MCQ, OSCE and DAS assessment; among 35 control group participants, all completed the post-intervention MCQ and DAS, but only 30 completed the post-intervention OSCE. All participants in Arm 1 who completed the SRCC also participated in clinical observations and interviews.

Generally, physicians who participated in the study were in their early 30s, primarily female and Malay (Table 2). Most physicians in all study arms were approximately the same age and had similar work experience, including experience in a primary health clinic as well as managing patients with diabetes.

**Table 2.** Distribution of physicians by demographic profile and work experience (n = 29)

Arm	1 (n = 9)	2 (n = 6)	3 (n = 8)	4 (n = 6)	*p*
**Age**					
Mean ± SD	33.27 ± 4.22	34.07 ± 4.39	32.41 ± 4.87	32.46 ± 1.94	0.885^α^
**Sex**					
Male (%)	3 (33.3)	2 (33.3)	2 (25.0)	1 (16.7)	0.023^Ω^
Female (%)	6 (66.7)	4 (66.7)	6 (75.0)	5 (83.3)	
**Ethnicity**					
Malay (%)	5 (55.6)	4 (66.7)	7 (87.5)	3 (50.0)	<0.001^Ω^
Chinese (%)	1 (11.1)	-	-	-	
Indian (%)	3 (33.3)	2 (33.3)	1 (12.5)	3 (50.0)	
**Working experience (years)**					
Mean ± SD	5.61 ± 2.15	6.66 ± 3.42	5.96 ± 4.63	7.32 ± 1.93	0.482^β^
Median	5.00	6.87	4.96	7.31	
**Experience at primary health clinic (months)**					
Mean ± SD	24.67 ± 24.26	42.17 ± 19.60	31.50 ± 24.31	51.83 ± 24.69	0.098 ^α^
Median	8	34	24	59	
**Experience working with diabetes patients (months)**					
Mean ± SD	34.78 ± 25.09	56.17 ± 45.11	31.50 ± 22.82	47.33 ± 36.11	0.417 ^α^
Median	42.0	28.5	35.0	50.0	

Data are expressed as mean ± SD, median or n (%); Significant difference between arms was determined by One-Way ANOVA ^α^, Chi-Square ^Ω^ or Kruskal-Wallis Test ^β^ at 0.05 level of significance.

The demographic profile and working experiences of nurses were similar to those of the physicians. However, most nurses had less working experience in primary care clinics and with managing or interacting with diabetes patients (Table 3). There were also no differences in age, duration of overall working experience and experiences in health clinics and with diabetes patients between nurses in the four study arms.

**Table 3.** Distribution of nurses by demographic profile and work experience (n = 40)

Arm	1 (n = 10)	2 (n = 9)	3 (n = 11)	4 (n = 10)	*p*
**Age**					
Mean ± SD	30.26 ± 8.8	28.39 ± 4.77	26.63 ± 1.67	30.93 ± 9.46	0.725
Median	26.93	27.76	26.75	28.59	
**Sex**					
Female (%)	10 (100.0)	9 (100.0)	11 (100.0)	10 (100.0)	
**Ethnicity**					
Malay (%)	10 (100.0)	9 (100.0)	7 (63.6)	10 (100.0)	<0.001 ^Ω^
Chinese (%)	-	-	1 (9.1)	-	
Indian (%)	-	-	3 (27.3)	-	
**Working experience (years)**					
Mean ± SD	7.58 ± 8.04	5.38 ± 4.15	3.38 ± 1.43	7.11 ± 9.0	0.803
Median	5.07	6.00	3.46	4.90	
**Experience in outpatient department at health clinic (months)**					
Mean ± SD	37.60 ± 60.85	26.78 ± 19.23	28.73 ± 14.01	33.10 ± 42.76	0.802
Median	13.0	19.0	36.0	19.5	
**Experience working with diabetes patients (months)**					
Mean ± SD	30.80 ± 27.84	26.0 ± 19.72	27.64 ± 11.66	33.00 ± 42.82	0.926
Median	27.5	19.0	36.0	19.0	

Data are expressed as mean ± SD, median or n (%); Significant difference between arms was determined by Chi-Square ^Ω^ or Kruskal-Wallis Test at 0.05 level of significanceķ.

### Impact of SRCC on knowledge, skills and attitudes

For both physicians and nurses, mean post-test scores for the MCQ and OSCE were higher in the intervention groups (Arms 1 and 2) compared to the control groups (Arms 3 and 4). However, no differences were observed for any of the five DAS-3 subscales between the intervention and control arms for either physicians or nurses (Table 4).

**Table 4.** Distribution of participants by post-intervention MCQ, OSCE and DAS results

Arm	1	2	3	4
Group	Intervention	Intervention	Control	Control
Pre-test	Yes	No	Yes	No
**MCQ** (range: 0 to 100)				
Physicians	80.62 ± 9.65	84.00 ± 6.13	63.13 ± 5.51	62.50 ± 12.34
Nurses	55.80 ± 9.61	61.10 ± 4.26	46.45 ± 8.47	39.80 ± 9.58
**OSCE** (range: 1 to 5)				
Physicians	3.60 ± 0.34	3.78 ± 0.34	3.29 ± 0.54	3.38 ± 0.51
Nurses	3.16 ± 0.37	3.07 ± 0.27	2.61 ± 0.36	2.80 ± 0.44
**DAS** (range: 1 to 5)				
Sub Scale 1 – Need for Special Training				
Physicians	4.20 ± 0.58	4.33 ± 0.33	4.60 ± 0.34	4.57 ± 0.29
Nurses	4.34 ± 0.28	4.31 ± 0.44	4.42 ± 0.45	4.33 ± 0.46
Sub Scale 2 – Seriousness of T2DM				
Physicians	4.01 ± 0.37	4.36 ± 0.48	4.27 ± 0.34	4.14 ± 0.30
Nurse	3.69 ± 0.36	3.67 ± 0.47	3.68 ± 0.28	3.65 ± 0.43
Sub Scale 3 – Value of tight control				
Physicians	3.79 ± 0.17	3.93 ± 0.32	3.80 ± 0.30	4.00 ± 0.36
Nurses	3.14 ± 0.51	3.08 ± 0.39	3.55 ± 0.44	3.38 ± 0.48
Sub Scale 4 – Psychosocial Impact of DM				
Physicians	4.00 ± 0.50	4.14 ± 0.27	4.23 ± 0.49	4.06 ± 0.23
Nurses	3.62 ± 0.39	3.89 ± 0.61	3.89 ± 0.63	3.50 ± 0.40
Sub Scale 5 – Patient Autonomy				
Physicians	3.92 ± 0.50	4.23 ± 0.39	3.92 ± 0.42	3.81 ± 0.49
Nurse	3.88 ± 0.27	3.72 ± 0.30	3.70 ± 0.33	3.83 ± 0.38

Data are expressed as mean ± SD.

The main effects of intervention on knowledge, skills and attitudes were analysed using Braver and Braver’s methodology (1988) [26]. Table 5 shows the results of the 2 × 2 factorial ANOVA on the mean post-test scores for knowledge (MCQ), skills (OSCE) and attitudes (DAS) for the four arms. The significant F-ratios on intervention effects for physicians on MCQ and OSCE scores showed that there were significant improvements after SRCC. Conversely, the non-significant F-ratios on interaction effects for physicians on MCQ and OSCE scores showed that pre-test exposure did not significantly affect observed improvements, confirming that increased knowledge and skills in diabetes care management among physicians could be attributed to the SRCC.

**Table 5.** Variance on post-test scores for MCQ, OSCE and DAS for physicians and nurses

	Physicians		Nurses	
	F	*p*	F	*p*
**MCQ**				
Intervention Effect	34.493	<0.001	33.412	<0.001
Interaction Effect (Pretest X Intervention)	0.363	0.553	5.090	0.030
**OSCE**				
Intervention Effect	4.656	0.042	11.030	0.002
Interaction Effect (Pretest X Intervention)	0.025	0.876	1.321	0.259
**DAS**				
**Sub-scale 1 – Need for Special Training**				
Intervention Effect	4.038	0.056	0.143	0.708
Interaction Effect (Pretest X Intervention)	0.280	0.602	0.044	0.834
**Sub-scale 2 – Seriousness of T2D**				
Intervention Effect	0.015	0.902	0.011	0.916
Interaction Effect (Pretest X Intervention)	1.872	0.184	0.000	0.982
**Sub-scale 3 – Value of tight control**				
Intervention Effect	0.162	0.691	5.689	0.023
Interaction Effect (Pretest X Intervention)	0.058	0.811	0.117	0.734
**Sub-scale 4 – Psychosocial Impact of DM**				
Intervention Effect	0.216	0.647	0.110	0.742
Interaction Effect (Pretest X Intervention)	0.990	0.330	3.927	0.055
**Sub-scale 5 – Patient Autonomy**				
Intervention Effect	1.446	0.241	0.082	0.777
Interaction Effect (Pretest X Intervention)	1.446	0.241	1.836	0.184

Participating nurses also showed a significant improvement on OSCE scores after SRCC, even after taking pre-test exposure into account. However, the 2 × 2 ANOVA did not clearly indicate whether significant improvements in nurses’ MCQ scores were due to the SRCC alone, due to the presence of a significant interaction effect (Table 5). Consequently, further analysis was performed on nurses’ MCQ post-test scores in the pretested and un-pretested groups by using a simple main effect test (Table 6). Significant findings in both pre-tested and un-pretested groups showed that the intervention had an effect on the improvement in nurses’ MCQ scores, even among un-pretested participants.

**Table 6.** Main effect test on the post-MCQ scores of nurses

Group	F	p
Pretested Group (Arm 1 and Arm 3)	5.614	0.029
Un-pretested Group (Arm 2 and Arm 4)	37.684	<0.001

The analysis of five DAS-3 subscales yielded more complex findings. In the initial 2 × 2 ANOVA analysis (Table 5), a significant intervention effect was observed for DAS-3 sub-scale 3 (value of tight control) for nurses. No other intervention effect in the remaining sub-scales for either physicians or nurses were statistically significant, nor were there interaction effects for the five subscales for either physicians or nurses. Further analysis consisted of assessing the intervention effect while adjusting for pre-test scores. It showed significant effects of SRCC for sub-scale 1 (need for training) for physicians and sub-scale 4 (psychosocial impact) for nurses.

The additional analysis of DAS-3 indicated that SRCC did not contribute to significant changes in diabetes-related attitudes among physicians, with the exception of their attitude towards the need for special training. Physicians in the intervention group had a lower mean score (Table 4), indicating a perceived need for training. Despite significant results for sub-scale 3 (value of tight control) and sub-scale 4 (psychosocial impact), nurses in the intervention groups, particularly those from Arm 1, had lower mean post-test scores, indicating a less positive attitude compared to their counterparts in the control groups (Table 4). Consequently, the effect of SRCC on attitudes as measured by the DAS-3 is unclear.

### Impact of SRCC on clinical practice

#### Observations

Analyses using repeated measure ANOVA on the two domains of soft skills and clinical skills showed significant improvements for both physicians and nurses in the intervention group (Table 7).

**Table 7.** Changes in clinical practices (transformed qualitative data)

	Observation							
	Soft skills				Clinical skills			
	Mean pre	Mean Post	∆	*p*	Mean pre	Mean Post	∆	*p*
Physicians	4.56 ± 0.73	4.89 ± 0.60	0.33 ± 0.50	<0.001	4.0 ± 0.71	5.11 ± 1.17	1.11 ± 0.78	<0.001
Nurses	3.00 ± 0.94	4.30 ± 1.25	1.30 ± 1.06	<0.001	2.3 ± 0.48	4.20 ± 1.32	1.90 ± 1.10	<0.001

Data are expressed as mean ± SD; Significant difference between arms was determined by repeated measures ANOVA at 0.05 level of significance.

Observations revealed that the majority of both physicians and nurses in Arm 1 had improved their soft skills in patient engagement by showing interest in patients’ concerns, providing opportunities for patients to express their thoughts and views and acknowledging and responding to patients’ perspectives. Excerpts from observation field notes support these findings.

*“One of the nurses who had just graduated from the nursing college hardly had any eye contact, conversation and interaction with the patients at the screening counter during the pre-intervention assessment. After completing SRCC, she was able to initiate conversation and conduct comprehensive history taking during the post-intervention assessment.”* (Nurse, 23 years old)

*“A nurse attached to the health clinic in a rural area was found to be paying more attention to patients’ issues and responded to their concerns accordingly (post-intervention).”* (Nurse, 27 years old)

In terms of clinical skills, most physicians in Arm 1 showed improvements in foot care, initiating and demonstrating insulin use and providing guidance on self-monitoring of blood glucose (SMBG). Most nurses in Arm 1 showed increases in providing basic information on nonpharmacological treatments, such as advice about diet and exercise, to patients at the screening counter. Several nurses also provided proper foot examination and advice on foot care or helped patients with SMBG. In addition, history taking substantially improved among all nurses.

*“A more senior nurse was noticed incorporating proper foot examination into the routine screening process. Advice on foot care such as types of footwear and socks, self-examination or feet, types of moisturisers to be used, avoidance of hot foot baths was also provided.”* (Nurse, 45 years old)

*“A newly graduated nurse, just attached at the clinic, indicated she had limited knowledge of diabetes care during the pre-intervention interview and was able to explain the pathophysiology of diabetes, the importance of urine test or other screening tests such as fundus examination, HbA_1C_, and provided basic information of the test results in a simple language or through illustrations to elderly patients during the post-intervention observation.”* (Nurse, 23 years old)

*“During the pre-intervention assessment, a nurse only performed blood pressure, finger-stick glucose check and weight measurement for diabetes patients who came for their follow-up visits. During the post-intervention assessment, a patient who was newly started on insulin brought her SMBG record and was referred to this nurse at the screening counter. The nurse reviewed the record and assisted her in titration to achieve the target fasting blood glucose.”* (Nurse, 23 years old)

#### Interviews

In interviews, all participants reported that they were able to practice some of their new knowledge and skills in their daily clinical work with diabetes patients (Table 8).

**Table 8.** Application of new knowledge by participants in diabetes care and other areas (qualitative interviews)

	Self-reported by participants			
	Diabetes care		Other areas	
	Yes	(%)	Yes	(%)
Doctors	9	100	7	77.8
Nurses	10	100	2	20

Excerpts from the interview transcripts, translated into English from colloquial Malay language, support these findings.

“*I initially thought I would not be able to apply any new knowledge or skills. However, surprisingly I did. I used the confidence ruler with an educated patient, and I felt it was a very interesting tool. I also made an agreement with a patient on a weight reduction target, and that made the patient more motivated to take action and show results at the next appointment*.” (Physician, 31-year-old female)

*“Yes, I think I am able to practice it. Even during the training and following the weekend workshops, I was able to change my style of counselling with my patients. Yes, I think what I have learned is very useful*.” (Physician, 30-year-old female)

Most nurses also indicated that they were able to practice what they had learned in SRCC, such as assisting patients with SMBG, providing accurate information on diet, oral therapy, insulin therapy, managing hypoglycaemia and demonstrating the correct technique for insulin injection and foot examinations in their daily tasks.

“*After completing the SRCC, the doctor referred to me patients for education on insulin self-titration. Now I have three patients under my responsibility, and I am confident of managing them. However, I will consult the doctor if I need assistance*.” (Nurse, 23 years old)

“*For clinical procedures, I understand and know more about conducting foot examinations*.” (Nurse, 29 years old)

Nonetheless, several participants felt that there were barriers to translating the knowledge and skills acquired in SRCC into action.

*“I cannot practice all that I have learned from SRCC sometimes because of time constraints. I also can’t apply the new knowledge and skills on all patients. I would focus on specific issues during a particular consultation, for example, weight management, sugar control or foot care, on what I think is important for the patient during that visit*.” (Physician, 30-year-old female)

In addition, most participants, particularly physicians, reported that they were able to apply the knowledge and skills they gained through SRCC to other areas or patients with other diseases, especially those with other NCDs (Table 8). Most stated that they were more aware of identifying individuals who had risk factors for NCDs, referred them for screening and early detection, prevention, treatment and empowered them to manage their own diseases.

*“I will be more attentive to conduct opportunistic screening for diabetes for patients attending the clinic, especially if they are obese or with risk factors for diabetes*.” (Physician, 30-year-old female)

*“I can apply what I learned from SRCC for patients with hypertension and heart diseases because we need to counsel them regarding adherence to medications and on lifestyle modification*.” (Physician, 30-year-old female)

*“We also manage pregnant patients diagnosed with diabetes and on insulin. However, for pregnant ladies with normal oral glucose tolerance test results, we sometimes don’t follow them up after delivery. After completing SRCC, I understand now the importance of annual screening post-delivery because these ladies are at high risk of developing diabetes*.” (Physician, 30-year-old female)

## Discussion

Physicians and nurses who completed the six-month SRCC showed significant improvements in diabetes care knowledge and skills. Both clinical and soft skills significantly improved for physicians and nurses alike. Participants reported that they were able to apply the new knowledge and skills in diabetes care and in other clinical areas. However, the findings did not support drawing clear conclusions about changes in participant’s attitudes about diabetes.

The ultimate goal of CME is to transfer learned knowledge, skills and attitude into the practice setting [27]. Research evaluating CME effectiveness has indicated that multiple educational platforms and case-based studies are more successful at improving patient health outcomes than are isolated traditional didactic sessions [28,29]. This evidence was the basic premise underlying the development and implementation of SRCC in Malaysia.

SRCC is unique in many ways. It emphasised self-driven learning with minimal supervision, using an e-learning platform, workbook and three-weekend workshops during the six-month training period. Programme delivery was designed to cause the least amount of disruption of service delivery at the health clinics and incur minimal costs to MOH.

CME is essential to ensuring highly qualified HCPs and achieving state-of-the-art clinical care for diabetes in particular and primary health care in general. However, it cannot stand alone. Other contextual factors are critical for HCPs to transfer the knowledge and skills to provide high-quality diabetes care, such as sufficient numbers of professional staff and adequate time for individual consultations, access to required equipment and skilful management and organisation of services at the clinic level. Furthermore, maintenance of acquired knowledge, skills and attitude through supervision and refresher courses is crucial, and the cost of initial and ongoing CME activities is important.

### Strengths and limitations

This study has two key strengths. The use of the Solomon four-group design enabled the researchers to assess the SRCC intervention effect separate from any confounding influence of pretest sensitisation. The study also applied mixed methods, which enabled a more comprehensive understanding of the intervention’s effects on knowledge, attitudes, skills and clinical practices.

Several limitations deserve mention. The study evaluated the SRCC alone, and it is not possible to assess how it compares to other training courses. Biases are also inherent in observational data collection methods, including the Hawthorne effect [30]. The use of interviews may have introduced the possibility of courtesy bias, which occurs when participants provide responses they think interviewers would like to hear. These biases can be mitigated through triangulation of data from different data collection methods [31]. The statistical analysis required transforming qualitative data about clinical practice into quantitative data. While we acknowledge the potential limitations of data transformation for statistical purposes, the process was clearly described before the assessment and only simple statistical measures of differences were used.

### Implication of findings

Currently, the SRCC curriculum targets only physicians and nurses. In keeping with a multi-disciplinary approach to diabetes care, the curriculum should be adapted for other HCPs involved with diabetes care in primary care, including assistant medical officers, dieticians, pharmacists and physiotherapists. Further evaluation studies of SRCC for other HCP categories should also be conducted. SRCC training for all HCPs in a health clinic is required to evaluate patient satisfaction and clinical outcomes as the ultimate impacts of SRCC on the quality of diabetes care.

Future research should investigate whether changes in observed and self-reported clinical practice translate into positive effects on clinical parameters such as HbA_1C_ and to evaluate the cost-effectiveness of the SRCC training programme. An opportunity also exists to use the SRCC learning methodologies to train HCPs in managing other chronic conditions commonly seen in primary care. NCDs necessitate the active involvement of patients in management, requiring HCPs to have adequate knowledge, skills and attitudes. Future research should evaluate the effect of adapting SRCC methodologies to other conditions or diseases in Malaysia. The robustness of the Solomon four-group design could also be examined in the evaluation of SRCC-based CME for other conditions.

## Conclusion

Significant improvements occurred in diabetes-related knowledge, skills and clinical practice among Malaysian physicians and nurses after participating in the six-month SRCC. The curriculum was designed specifically to meet the current needs of physicians and nurses working in primary care in Malaysia, and SRCC delivery caused minimal disruption to service delivery and used existing resources. SRCC thus constitutes an effective CME tool for improving clinical diabetes care that can be scaled up to the rest of the country and, with modification, beyond Malaysia. Furthermore, the SRCC CME methodology may be used for other groups of HCPs and to improve care for other noncommunicable diseases.
